# Supplementary material for: Unusual solute segregation phenomenon in coherent twin boundaries
Source: Nat Commun. 2021 Feb 1;12:722. doi: 10.1038/s41467-021-21104-8 (PMC7851144; doi:10.1038/s41467-021-21104-8)
Supplement: Supplementary file 1 — Supplementary Information [file 41467_2021_21104_MOESM1_ESM.pdf]

Supplementary Information

Unusual solute segregation phenomenon in coherent twin boundaries

Cong He, Zhiqiao Li, Houwen Chen*, Nick Wilson, J.F. Nie*

*Corresponding authors
hwchen@cqu.edu.cn (H.W. Chen), jianfeng.nie@monash.edu (J.F. Nie)

The PDF file includes:

Supplementary Text
Supplementary Figure 1. EDS results of a $\{10\bar{1}1\}$ twin boundary
Supplementary Figure 2. HAADF-STEM image simulations.
Supplementary Figure 3. Differential electron charge density (DECD) analysis for $\{10\bar{1}2\}$ CTBs.
Supplementary Figure 4. Three-dimensional isosurface patterns of the differential charge density contours for Bi-segregated $\{10\bar{1}1\}$ CTBs.
Supplementary Figure 5. Segregation energies of (a) Pb, (b) Tl and (c) In at compression or extension sites in $\{10\bar{1}1\}$ and $\{10\bar{1}2\}$ CTBs.
Supplementary Figure 6. Contour maps of DECD of Pb segregated into CTBs.
Supplementary Figure 7. Characterization of the solute segregation phenomenon in CTBs in a Mg–1.5Pb alloy.
Supplementary Figure 8. Absence of evident segregation of Y atoms in $\{11\bar{2}1\}$ CTBs.
Supplementary Figure 9. Supercells of $\{10\bar{1}1\}$ and $\{10\bar{1}2\}$ used in this work.
Supplementary Figure 10. Atomic model of $\{11\bar{2}1\}$ CTB in Mg.
Supplementary Figure 11. Twin boundary site geometries.
Supplementary Table 1. Some physical properties of solute elements involved in this work.
Supplementary Table 2. Bader analysis for solutes of Pb, Tl and In located in CTBs.
Supplementary Table 3. Segregation energy of Y substituting Mg at some atomic sites in $\{11\bar{2}1\}$ CTB.
Supplementary Table 4. Bader charge values of Y atoms segregated to the different sites of $\{11\bar{2}1\}$ CTB.
Supplementary References.

Supplementary Note 1

1. Characterization of CTBs in Mg–0.4Bi (at.%) alloy

The HAADF-STEM image in Supplementary Fig. 1a shows the atomic structure of a $\{10\bar{1}1\}$ TB in a sample of Mg–0.4Bi (at.%) alloy that was compressed by 11% and then aged at 80 °C for 48 h. STEM-EDS map in Supplementary Fig. 1b shows the element distribution of Bi atoms and it clearly reveals solute segregation of Bi atoms is detected in the $\{10\bar{1}1\}$ TB.

To evaluate the solute occupancy of Bi atoms in a single Bi-rich column in the HAADF-STEM image of the $\{10\bar{1}1\}$ CTB shown in Fig. 1, image simulation using xHREM software package[1] was applied. This method has been widely used to obtain the quantitative occupancy of solute atoms in each column according to image intensity analysis[2,3]. The solute occupancy series of Bi atoms including 20 at.%, 40 at.%, 60 at.%, 80 at.%, and 100 at.% in a single Bi-rich column were adopted when performing image simulations. The foil thickness is 75 nm (determined by PACBED). Simulated HAADF-STEM images with different Bi atom occupancies are shown in Supplementary Figs. 2a-2e. Note that in the experimental image (Supplementary Fig. 2f), the average intensity of Mg atom columns is ~53% of the average intensity of bright Bi-rich columns. Comparison image intensities of sequential simulated images with that of the experimental image suggests that the experimental occupancy in a single Bi-rich column is estimated as ~20 at.%.

2. Differential electron charge density (DECD) of Bi, Gd, Zn segregated to CTBs

Supplementary Fig. 3 shows the DECD plane contour of Bi, Gd, Zn occupying compression sites and extension sites of $\{10\bar{1}2\}$ CTBs. From the DECD images, it is found that similar to the DECD patterns of solute segregation at $\{10\bar{1}1\}$ CTBs, solute Bi and Zn attract valence electrons from Mg matrix and Gd loses its valence electrons to nearby Mg atoms. However, according to Supplementary Fig. 3, the differences of charge distributions between solute occupying the compression sites and extension sites are not as obvious as that in $\{10\bar{1}1\}$ CTBs. The specific differences are well established through Bader charge analysis and their values are contained in Table 1.

Supplementary Fig. 4 shows the three-dimensional isosurface DECD patterns of Bi-segregated $\{10\bar{1}1\}$ CTBs. The orange and green balls represent Bi and Mg atoms, respectively. The yellow shell surrounding Bi atoms describe the space with relatively higher valence electron density. Clearly, the yellow shell is bigger when Bi atoms segregate at the compression site rather than at the extension site, indicating more valence electrons will be absorbed by Bi atom at the compression site of $\{10\bar{1}1\}$ CTBs.

3. Calculation results of other solute atoms of Pb, Tl and In

To prove that our findings are not merely appropriate for solute Bi, Gd and Zn, several other solutes such as Pb, Tl and In (Supplementary Table 1) that have larger atomic size, *p*-type valence electrons and similar electronegativity to Bi were also considered. The segregation energies at $\{10\bar{1}1\}$ and $\{10\bar{1}2\}$ CTBs with different occupancies of Pb, Tl and In atoms in a single column were calculated (Supplementary Fig. 5). For further analyzing, the DECD images of Pb (Supplementary Fig. 6) and the Bader charge values of Pb, Tl, In segregation (Supplementary Table 2) are also established. From Supplementary Fig. 5, it is found that Pb, Tl and In are very similar to solute Bi from the view of segregation energies, indicating their similar segregation behaviors. Moreover, the calculated DECD images of Pb at the compression site and extension site of $\{10\bar{1}1\}$ and $\{10\bar{1}2\}$ CTBs are almost the same as that of Bi (Supplementary Fig. 6). In Supplementary Table 1, the electronegativity values of Pb, Tl, In and Mg are 1.8, 1.8, 1.78 and 1.31, respectively, indicating that

Pb, Tl and In behave in similar chemical feature with Bi and Zn due to their higher electronegativity values than that of Mg. From the Bader charge values in Supplementary Table 2, a stronger chemical bond between the solute atom and the adjacent Mg atoms will be formed at the compressive sites of CTBs than that at the extension sites and that in Mg matrix. Simultaneously, Pb, Tl and In are larger than Mg in atomic radius. Thus, there is a competition between chemical bonding effect and elastic strain minimization when determining the segregation behaviors of these three solutes. Solute segregation of these atoms at CTBs are possible when the stabilizing effect from chemical bonding effect is strong enough to overcome the unstabilizing effect from intensified local strain field, e.g., the segregation at compression sites of $\{10\bar{1}1\}$ CTBs with 20% occupancy in a single column (Supplementary Fig. 5), or when the stabilizing effect brought by relieving local strain exceeds the unstabilizing effect from weakening chemical bonding, e.g., the segregation at tension sites of $\{10\bar{1}1\}$ CTBs with 20% occupancy (Supplementary Fig. 5). Such competition is verified in the Pb segregation behaviors at $\{10\bar{1}1\}$ and $\{10\bar{1}2\}$ CTBs in the Mg–1.5Pb (at.%) alloy, as shown in Supplementary Fig. 7.

4. Characterization of the CTBs in Mg–1.5Pb (at.%) alloy

Supplementary Fig. 7a shows the microstructure of a $\{10\bar{1}1\}$ CTB in Mg–1.5Pb (at.%) alloy after aged at 200 °C for 5 h. The bright dots along the CTB in this HAADF-STEM image indicate these columns are Pb-rich. According to the alternative distribution of compressive and extensive sites within the $\{10\bar{1}1\}$ CTB, Pb atoms that have a larger atomic radius than Mg atoms, unambiguously segregate into the contracted sites, as marked by the orange dashed-line circle. Since HAADF-STEM are unable to determine whether Pb atoms take 100% occupancy or partially occupied in a single Pb-rich column at CTBs, HAADF image simulation is applied to perform a nearly quantitative analysis. Our HAADF image simulation indicates that a solute occupancy of ~40 at.% Pb in a Pb-rich column leads to image intensities matching well with the corresponding experimental image.

The HAADF-STEM image in Supplementary Fig. 7b shows a $\{10\bar{1}2\}$ CTB in Mg–1.5Pb (at.%) alloy after aged at 200 °C for 5 h. Again, no apparent solute segregation of Pb atoms is found in the $\{10\bar{1}2\}$ CTB. This unusual segregation phenomenon at $\{10\bar{1}1\}$ and $\{10\bar{1}2\}$ CTBs in Mg–1.5Pb (at.%) alloy is very similar to the observations in the Mg–0.4Bi (at.%) alloy (Figs. 1 and 2).

5. Absence of Y segregation in $\{11\bar{2}1\}$ CTBs in Mg–2.0Y (at.%) alloy

$\{11\bar{2}1\}$ twins have been observed to form mainly in RE-containing Mg alloys, such as Mg–Y[6]. However, solute segregation is not observed in $\{11\bar{2}1\}$ CTBs in the Mg–2at%Y alloy that has been pre-compressed by 5% and then aged at 200 °C for 8 hours (Supplementary Fig. 8a) or even 17 hours (Supplementary Fig. 8b), although the extension strain along the $\{11\bar{2}1\}$ CTB[7] favors Y atoms, larger than Mg, to segregate to the extension sites. Note that Y segregation was detected in $\{10\bar{1}2\}$ CTBs, after ageing treatment for 8 and 17 hours at 200 °C (not shown). To test whether our computation analysis is applicable to the $\{11\bar{2}1\}$ twins, first-principles calculations were also carried out. Supplementary Fig. 10 is the atomic diagram showing the input $\{11\bar{2}1\}$ twin supercell. Three possible segregation sites Site 1-3 were considered. The segregation energy values of these sites are listed in Supplementary Table 3. All the values are in rather small scale, indicating that Y atoms have little segregation propensity in the $\{11\bar{2}1\}$ CTB, since most obvious solute segregation phenomena at CTBs correspond to a much more negative segregation energy value[5,8]. The Bader charge analysis was also further carried out, and the main results are provided in Supplementary Table 4. Bader charge values of Y atoms segregated to the three sites are all smaller than that located in the Mg matrix, which suggests that the chemical bonding effect is relatively weak. These results imply that our

conclusion (solute segregation dominated by strong chemical bonding effect) is capable of explaining why no obvious Y segregation was observed experimentally (Supplementary Fig. 8).

Supplementary figures and tables

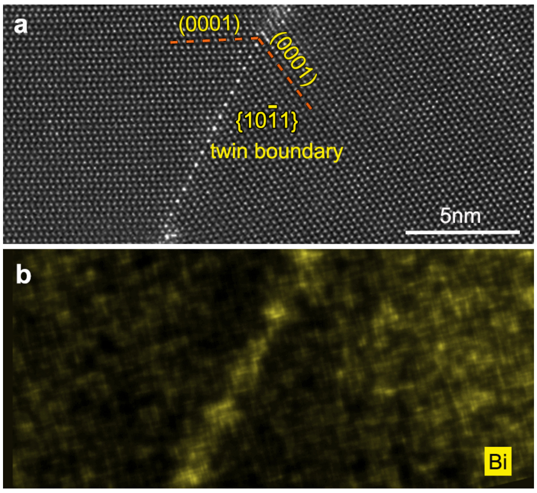

Supplementary Figure 1. EDS results of a {10$\bar{1}$1} twin boundary. (**a**) HAADF-STEM image showing a {10$\bar{1}$1} twin boundary in a sample of Mg–0.4Bi (at.%) alloy that was compressed by 11% and then aged at 80 °C for 48 hours. (**b**) STEM-EDS map showing Bi segregation in the twin boundary shown in (**a**). Electron beam direction is parallel to [1$\bar{2}$10].

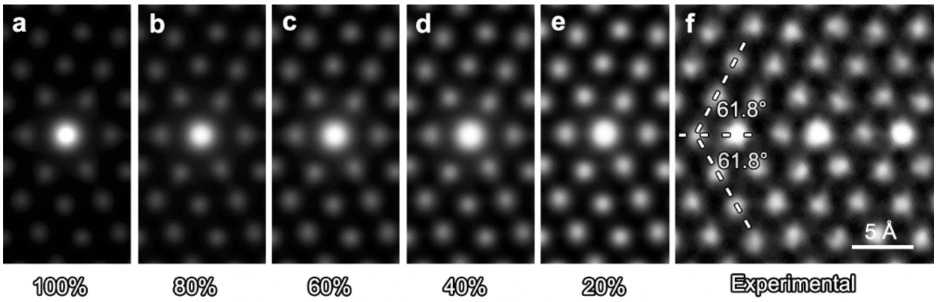

Supplementary Figure 2. HAADF-STEM image simulations. (**a-e**) Simulated HAADF-STEM images with different Bi atom occupancies showing the constructed model from the experimental $\{10\bar{1}1\}$ CTB shown in Fig. 1. The foil thickness is 75 nm (measured by PACBED). The value of Bi atom occupancies is (**a**) 100 at.%, (**b**) 80 at.%, (**c**) 60 at.%, (**d**) 40 at.%, and (**e**) 20 at.%. (**f**) Experimental HAADF-STEM image of the $\{10\bar{1}1\}$ CTB shown in Fig. 1.

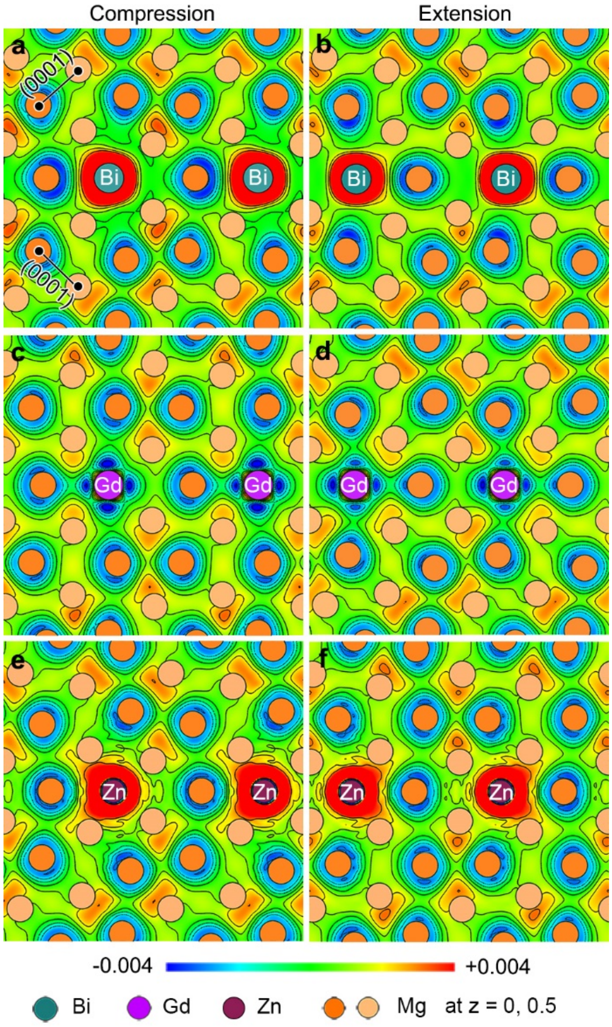

Supplementary Figure 3. Differential electron charge density (DECD) analysis for {10$\bar{1}$2} CTBs. Contour maps of differential electron charge density (DECD) of (**a**, **b**) Bi, (**c**, **d**) Gd and (**e**, **f**) Zn segregated in {10$\bar{1}$2} CTBs. (**a**), (**c**) and (**e**) correspond to solute segregation at compression sites while (**b**), (**d**) and (**f**) correspond to solute segregation at extension sites. The unit of scale bar is eV/Å^3.

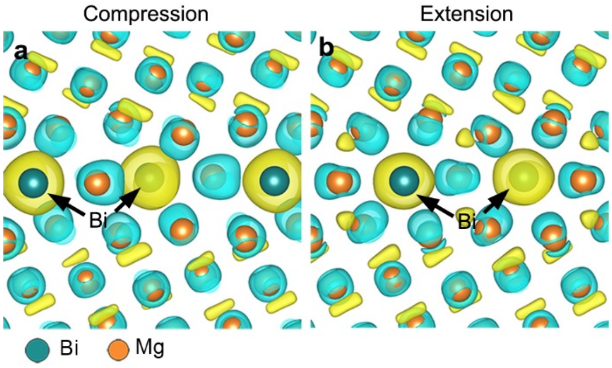

Supplementary Figure 4. Three-dimensional isosurface patterns of the differential charge density contours for Bi-segregated {10$\bar{1}$1} CTBs. Bi atoms segregate at (**a**) compression site or (**b**) extension site of {10$\bar{1}$1} CTBs. Bi atoms are highlighted by black arrows. The isosurface level value is 0.0025 when generating these plots.

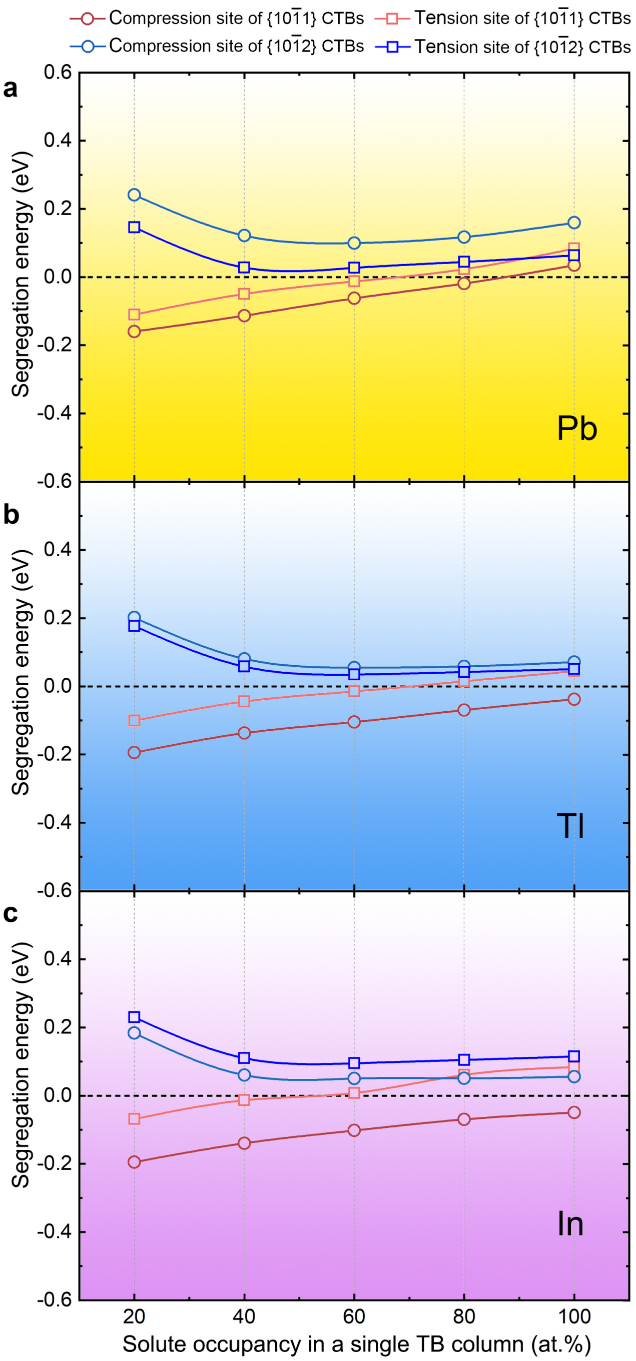

Supplementary Figure 5. Segregation energies of (a) Pb, (b) Tl and (c) In at compression or extension sites in {10$\bar{1}$1} and {10$\bar{1}$2} CTBs.

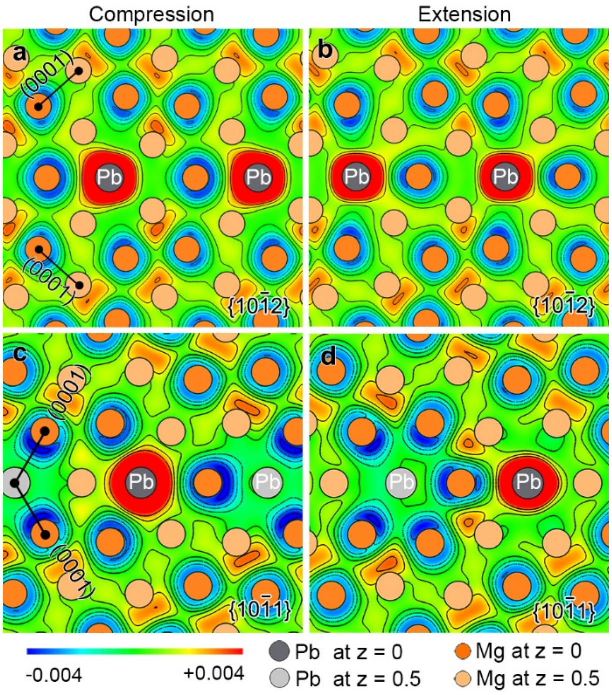

Supplementary Figure 6. Contour maps of DECD of Pb segregated into CTBs. (**a**, **b**) $\{10\bar{1}2\}$ CTBs and (**c**, **d**) $\{10\bar{1}1\}$ CTBs of $(11\bar{2}0)$. Solute Pb atoms segregate to (**a**, **c**) the compression sites and (**b**, **d**) the extension sites CTBs. The unit of scale bar is eV/Å^3.

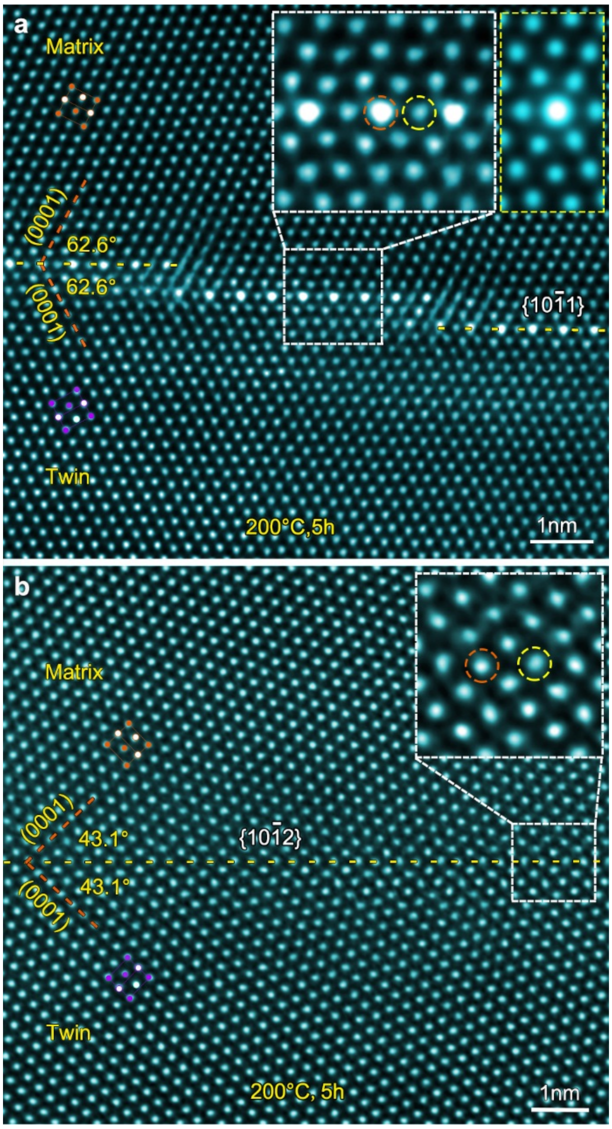

Supplementary Figure 7. Characterization of the solute segregation phenomenon in CTBs in Mg–1.5Pb alloy. (**a**) HAADF-STEM image showing Pb segregation in a $\{10\bar{1}1\}$ CTB in a sample in Mg–1.5Pb alloy compressed by 17% and then aged at 200 °C for 5 hours. An enlargement of a local region of this CTB is shown in the upper-right inset, indicating that Pb atoms segregate to compression sites of the $\{10\bar{1}1\}$ CTB. Electron beam direction is parallel to $[1\bar{2}10]$. Simulated HAADF image with a foil thickness of ~100 nm (measured by PACBED) is inserted and enclosed by a yellow dotted-line rectangular frame. (**b**) HAADF-STEM image showing the absence of Pb segregation in a $\{10\bar{1}2\}$ CTB in Mg–1.5Pb alloy compressed by 9% and then aged at 200 °C for 5 hours. Enlarged image of this CTB reveals that no Pb-rich columns are detected. The alternate compression and extension sites of the CTB are marked by orange and yellow dash-line circles, respectively.

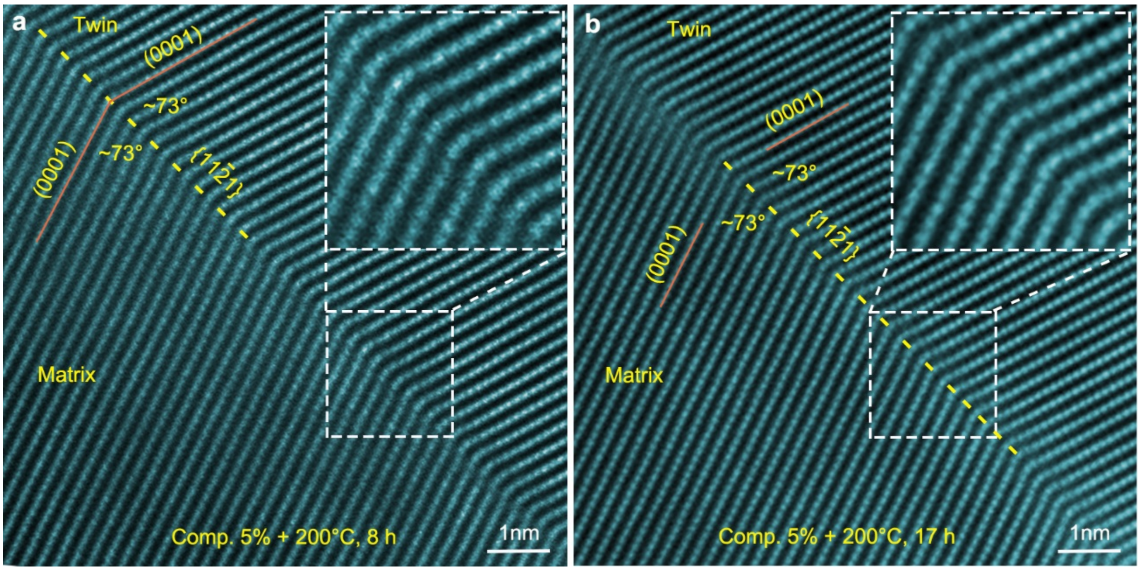

Supplementary Figure 8. Absence of evident segregation of Y atoms in {11$\bar{2}$1} CTBs. HAADF-STEM images showing {11$\bar{2}$1} CTBs in the sample of Mg–2at.%Y alloy which was compressed by 5% and then aged for (**a**) 8 hours or even (**b**) 17 hours at 200 °C.

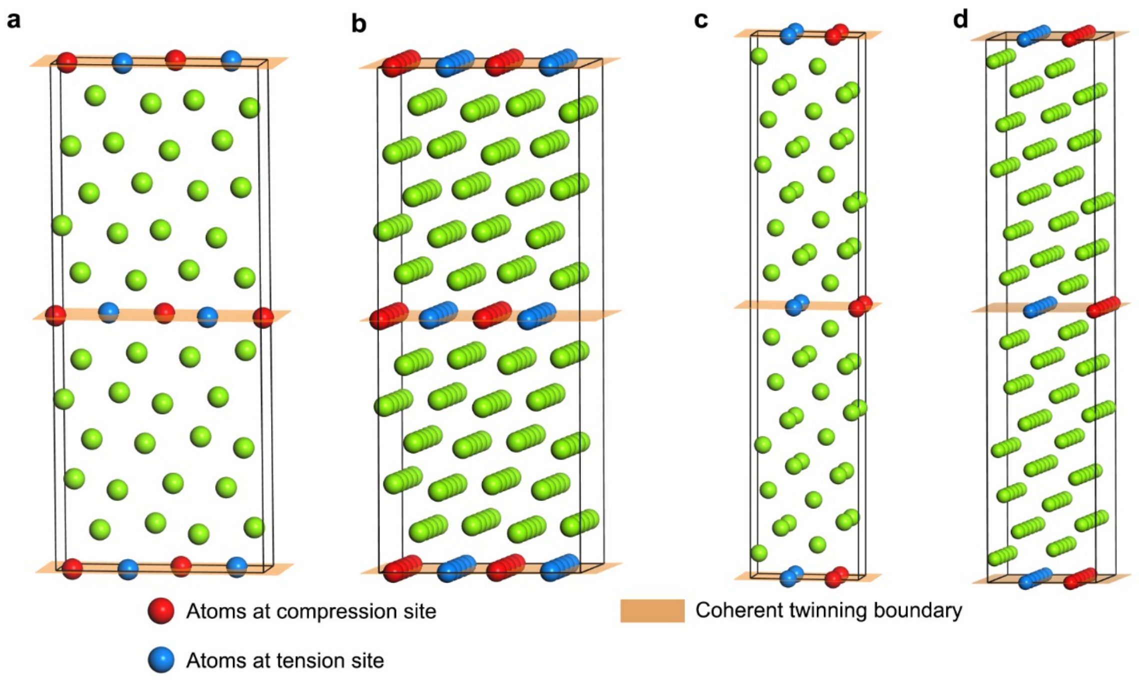

Supplementary Figure 9. Supercells of {10$\bar{1}$1} and {10$\bar{1}$2} used in this work. (**a**) {10$\bar{1}$1}, 1 × 1 × 1, 48 atoms. (**b**) {10$\bar{1}$1}, 1 × 5 × 1, 240 atoms (**c**) {10$\bar{1}$2}, 1 × 1 × 1, 40 atoms. (**d**) {10$\bar{1}$2}, 1 × 5 × 1, 200 atoms. Atoms at the compression and extension sites of {10$\bar{1}$1} and {10$\bar{1}$2} CTBs are represented by red and blue balls. The orange planes highlight the CTBs locating at the middle and bottom of the supercells.

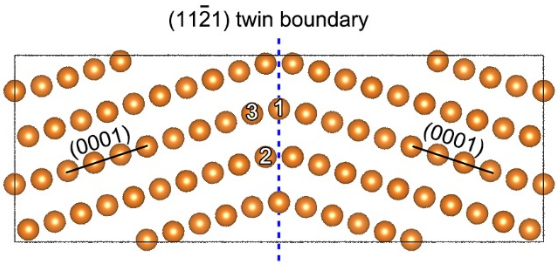

Supplementary Figure 10. Atomic model of {11$\bar{2}$1} CTB in Mg. Y atom was embedded at the sites of {11$\bar{2}$1} CTB to calculate the corresponding segregation energies, as marked by numbers 1-3.

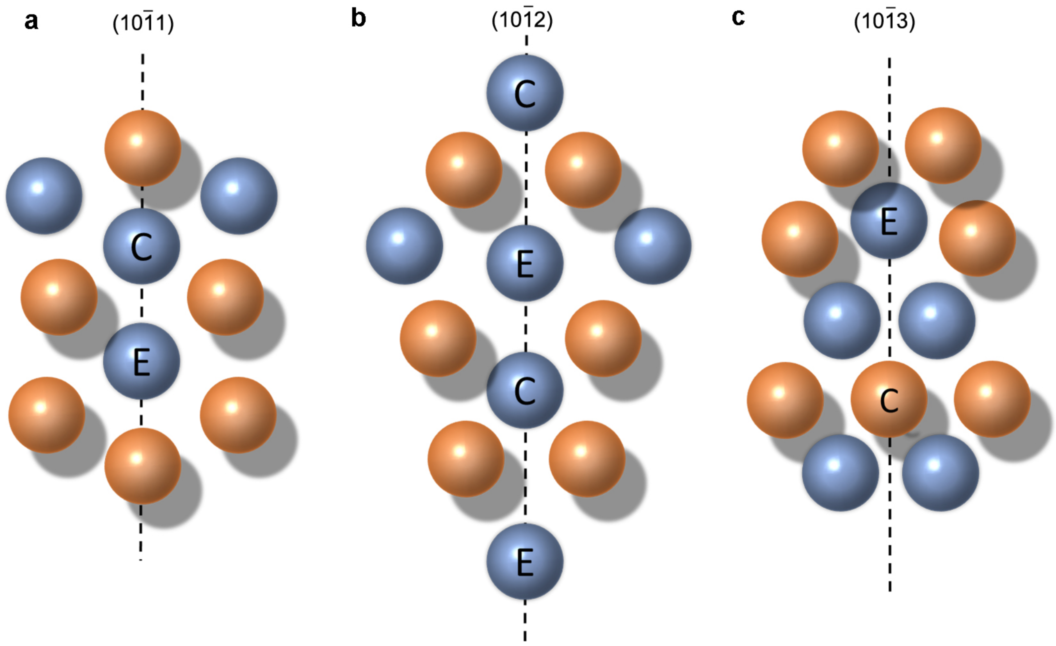

Supplementary Figure 11. Twin boundary site geometries. Extension and compression sites, labelled E and C along (**a**) the $\{10\bar{1}1\}$ CTB, (**b**) the $\{10\bar{1}2\}$ CTB and (**c**) the $\{10\bar{1}3\}$ CTB. Mg atoms colored blue all sit in the same plane, while Mg atoms colored orange sit in the adjacent planes.

Supplementary Table 1. Some physical properties of solute elements involved in this work.

Solute element	Atomic number	Metallic radii / pm	Empirical radii / pm[c]	Electronegativity[d]	Electron configuration
Mg	12	160[a], 159.8[b]	150	1.31	[Ne]$3s^2$
Gd	64	180[a], 179.5[b]	180	1.20	[Xe]$4f^7 5d^1 6s^2$
Bi	83	–[a], 170[b]	160	1.9	[Hg]$6p^3$
Pb	82	–[a], 174.6[b]	180	1.8	[Hg]$6p^2$
Tl	81	170[a], 171.2[b]	190	1.8	[Hg]$6p^1$
In	49	167[a], 166.0[b]	155	1.78	[Kr]$4d^{10} 5s^2 5p^1$
Zn	30	134[a], 137.9[b]	135	1.65	[Ar]$3d^{10} 4s^2$

[a] Ref.[9]
[b] Ref.[10]
[c] Ref.[11]
[d] Ref.[12]

Supplementary Table 2. Bader analysis of solute Pb, Tl or In located in CTBs. Bader charge values (*e*) of Pb, Tl or In atom located in the Mg matrix and occupying compression or extension sites of $\{10\bar{1}1\}$, compression or extension sites of $\{10\bar{1}2\}$ CTBs. The cases of 20% occupancy are also listed.

Solute	Matrix	Comp. sites of $\{10\bar{1}1\}$ CTBs	Ext. sites of $\{10\bar{1}1\}$ CTBs	Comp. sites of $\{10\bar{1}2\}$ CTBs	Ext. sites of $\{10\bar{1}2\}$ CTBs
Pb (100% occupancy)	6.08*	5.82	5.13	5.63	4.95
Pb (20% occupancy)	–	6.30	5.71	6.35	5.58
Tl (100% occupancy)	4.87*	4.85	4.04	4.67	3.86
Tl (20% occupancy)	–	5.19	4.50	5.24	4.40
In (100% occupancy)	5.21*	5.16	4.24	4.93	4.09
In (20% occupancy)	–	5.49	4.77	5.63	4.64

*calculated from embedding a single solute atom in Mg matrix.

Supplementary Table 3. Segregation energy of Y substituting Mg at some atomic sites in {11$\bar{2}$1} CTB.

Site	Segregation energy (eV)
1	0.0056
2	-0.0147
3	-0.0074

Supplementary Table 4. Bader charge values (*e*) of Y atoms segregated to different sites of $\{11\bar{2}1\}$ CTB.

Site	Bader charge (*e*)
1	10.45
2	10.43
3	10.45
Matrix	10.47

Supplementary references

1. Ishizuka, K. A practical approach for STEM image simulation based on the FFT multislice method. *Ultramicroscopy* **90**, 71-83 (2002).
2. Yan, Y. et al. Impurity-induced structural transformation of a MgO grain boundary. *Phys. Rev. Lett.* **81**, 3675-3678 (1998).
3. Zhao, X. et al. Edge segregated polymorphism in 2D molybdenum carbide. *Adv. Mater.* **31**, 1808343 (2019).
4. Li, J. AtomEye: an efficient atomistic configuration viewer. *Modell. Simul. Mater. Sci. Eng.* **11**, 173 (2003).
5. Nie, J. F., Zhu, Y. M., Liu, J. Z. & Fang, X. Y. Periodic segregation of solute atoms in fully coherent twin boundaries. *Science* **340**, 957 (2013).
6. Stanford, N., Marceau, R. K. W. & Barnett, M. R. The effect of high yttrium solute concentration on the twinning behaviour of magnesium alloys. *Acta Mater.* **82**, 447-456 (2015).
7. He, C. et al. Origin of profuse $\{11\bar{2}1\}$ deformation twins in Mg-Gd alloys. *Scr. Mater.* **191**, 62-66 (2021).
8. Zhang, J., Dou, Y. & Zheng, Y. Twin-boundary segregation energies and solute-diffusion activation enthalpies in Mg-based binary systems: a first-principles study. *Scr. Mater.* **80**, 17-20 (2014).
9. Greenwood, N. N. & Earnshaw, A. *Chemistry of the Elements*. (Butterworth-Heinemann, UK, 1997).
10. Pauling, L. Atomic radii and interatomic distances in metals. *J. Am. Chem. Soc.* **69**, 542-553 (1947).
11. Slater, J. C. Atomic radii in crystals. *J Chem. Phys.* **41**, 3199-3204 (1964).
12. Lide, D. R. *Handbook of Chemistry and Physics, 84th Edition*. (CRC Press, Boca Raton, 2003).
